# Otogenic Brain Abscess: Judicious Management in a Case of Chronic Suppurative Otitis Media

**DOI:** 10.7759/cureus.30430

**Published:** 2022-10-18

**Authors:** Senu Sunnychan, Prasad Deshmukh, Sagar S Gaurkar, Arjun Panicker, Aishwarya Vijayappan

**Affiliations:** 1 Otolaryngology-Head and Neck Surgery, Jawaharlal Nehru Medical College, Datta Meghe Institute of Medical Sciences, Wardha, IND; 2 Otolaryngology-Head and Neck Surgery/Surgical Oncology, Jawaharlal Nehru Medical College, Datta Meghe Institute of Medical Sciences, Wardha, IND

**Keywords:** burr hole drainage, modified radical mastoidectomy, chronic suppurative otitis media, intracranial complication, cerebellar abscess, otogenic brain abscess

## Abstract

Intracranial consequences from chronic otitis media can be dreadful. Meningitis is the most frequent complication followed by a cerebral abscess. In this pre-antibiotic era, otogenic brain abscess is rare, but it poses one of the life-threatening complications of otitis media. In recent years, brain abscess was noticed almost only in patients of chronic Otitis media with cholesteatoma. A case of a 36-year-old non-diabetic male patient with an otogenic cerebellar abscess, who presented with no cerebellar signs and unique intraoperative ossicular chain status was successfully managed by a combined approach of otolaryngology and neurosurgery, is presented in this report.

## Introduction

Chronic otitis media (COM) is an inflammatory process in the middle-ear space that results in long-term, or more often, permanent changes in the tympanic membrane including atelectasis, perforation, tympanosclerosis, retraction pocket development, or cholesteatoma [1]. Long-term Eustachian tube dysfunction with a poorly aerated middle-ear space, multiple bouts of acute otitis media, persistent middle-ear infection, or other chronic inflammatory stimuli are some of the etiological factors of COM. The microbiological picture of COM shows the involvement of organisms such as *Pseudomonas aeruginosa*, very rare in acute otitis media.

Once the infection spreads beyond the confines of the lining mucoperiosteum of the middle-ear cleft, it opens door to various complications among which a brain abscess is being dealt with in this report [1]. Most of the authors have delineated the location of otogenic brain abscesses in the cerebrum (temporal lobe) than in the cerebellum; however, it's an interesting observation that majority of cerebellar abscesses are associated with middle ear infections [2]. Nevertheless, Murthy and Dubey found the occurrence of otogenic abscesses frequently in the cerebellum. Despite diagnostic and therapeutic achievements, mortality from otogenic brain abscesses is still relatively high and usually requires combined neurosurgical and otolaryngological surgery [2]. A case of otogenic brain abscess in a non-diabetic middle-aged male managed successfully by modified radical mastoidectomy and simultaneous abscess drainage via burr hole is reported here.

## Case presentation

**Figure 1:**
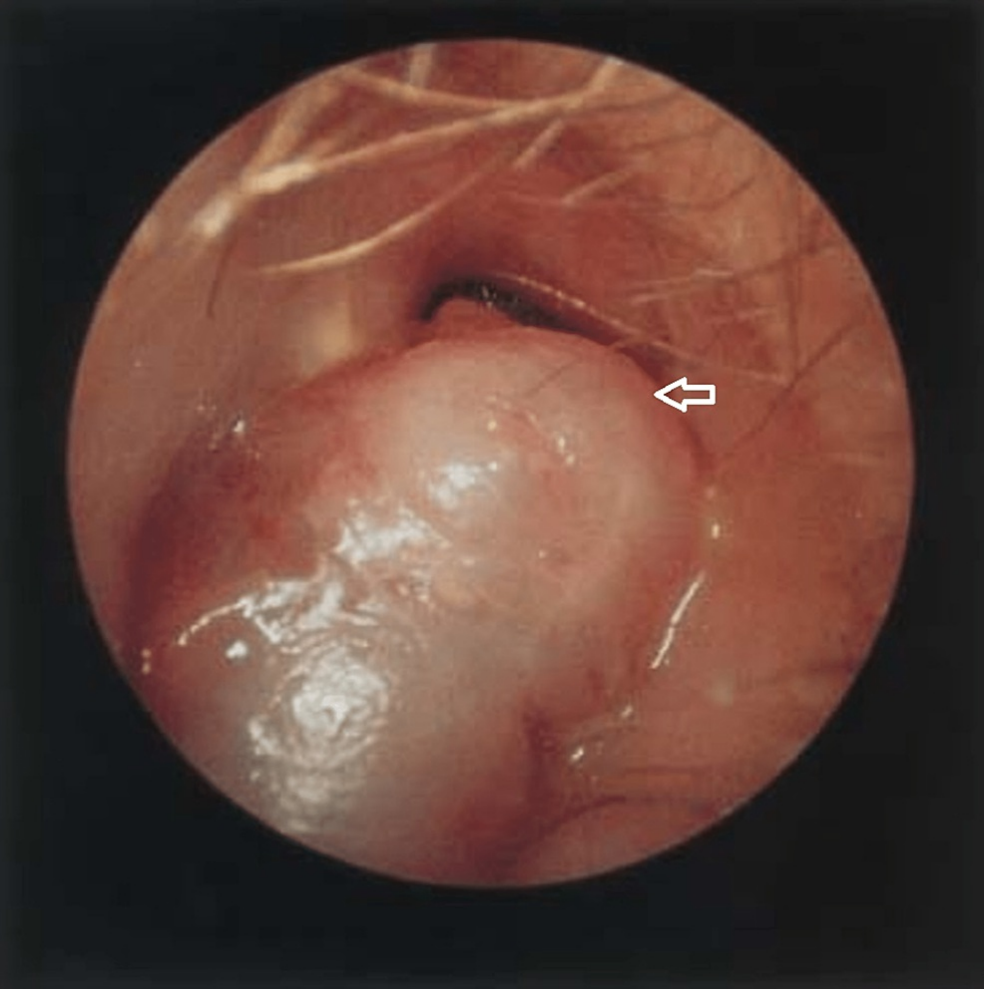
Microscopic examination image Examination under a microscope showing the right external auditory canal with fleshy mass (white arrow).

A 36-year non-diabetic male patient presented to the ENT clinic in Acharya Vinoba Bhave Hospital, Wardha, with complaints of headache, dizziness, and a sense of imbalance. The patient had intermittent right ear discharge for the past 16 years which used to subside with treatment. The present episode of ear discharge started one month back. It was a mucopurulent, foul-smelling, non-blood-stained discharge that did not reduce with medical management. The patient experienced a frontal headache for one month which suddenly progressed to severe in intensity to interfere with his daily activity for two days and was associated with dizziness and vomiting. He was afebrile and was oriented to time, place, and person. ENT examination showed a fleshy mass along with a thick greenish discharge in the right external auditory canal (Figure 1). Probing the mass showed attachment to the posterosuperior part of the external auditory canal which was non-friable and did not bleed. Vestibular functions were unaltered. The central nervous system examination was normal. Routine blood investigations showed an elevated total WBC count with an elevated erythrocyte sedimentation rate (ESR) and increased neutrophil count.

**Figure 2:**
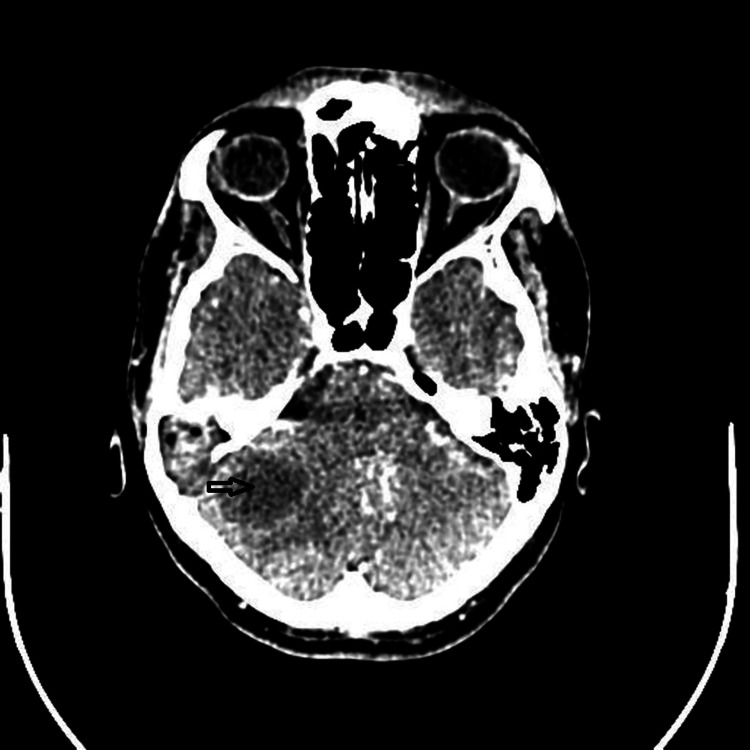
CT brain (axial section) The arrow shows hypodensity in the right hemisphere of the cerebellum

The patient was instructed to undergo a CT (computed tomography) scan which identified the patient's condition as chronic suppurative otitis media with soft tissue mass in the external ear, middle ear, and mastoid eroding the tegmen tympani with a peripherally increased hypodensity in the right hemisphere of the cerebellum measuring approximately 4 x 3 cm (Figure 2). 

**Figure 3:**
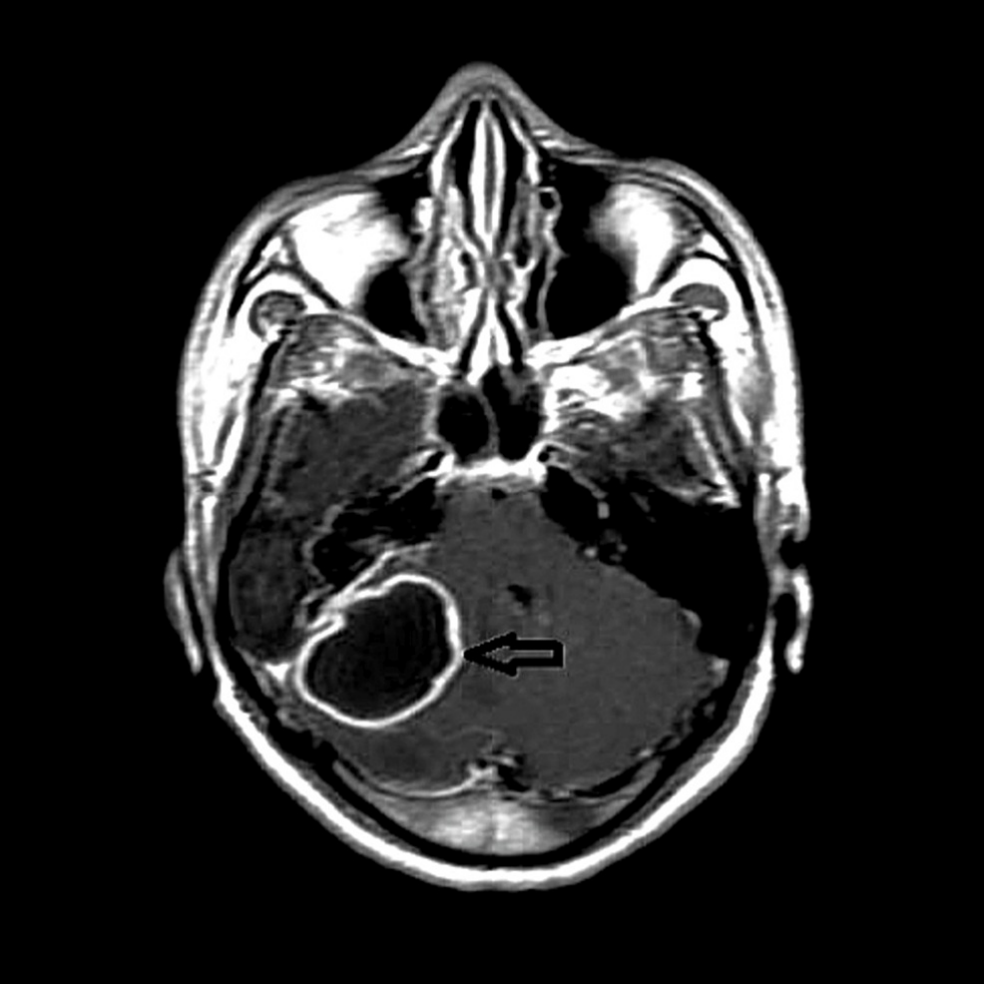
T2 weighted MRI brain (axial section) The image is showing a peripherally rim-enhancing altered signal intensity lesion in the right cerebellar hemisphere (arrow).

MRI (magnetic resonance imaging ) brain with contrast suggested cholesteatoma formation in the right external ear, middle, and mastoid eroding the tegmen tympani with evidence of peripherally rim enhancing altered signal intensity lesion in the right cerebellar hemisphere appearing hyperintense on T2, hypointense on T1 /FLAIR (fluid-attenuated inversion recovery) showing restriction on DWI (diffusion-weighted imaging ) and dark signals on ADC (apparent diffusion coefficient), measuring approximately 38 x 26 x 25 mm with perilesional oedema. The lesion was causing a mass effect in the form of effacement of the ipsilateral cerebellar hemisphere, 4th ventricle, right cerebellar peduncle and pons and midbrain on the right side with right sigmoid sinus thrombosis (Figure 3).

**Figure 4:**
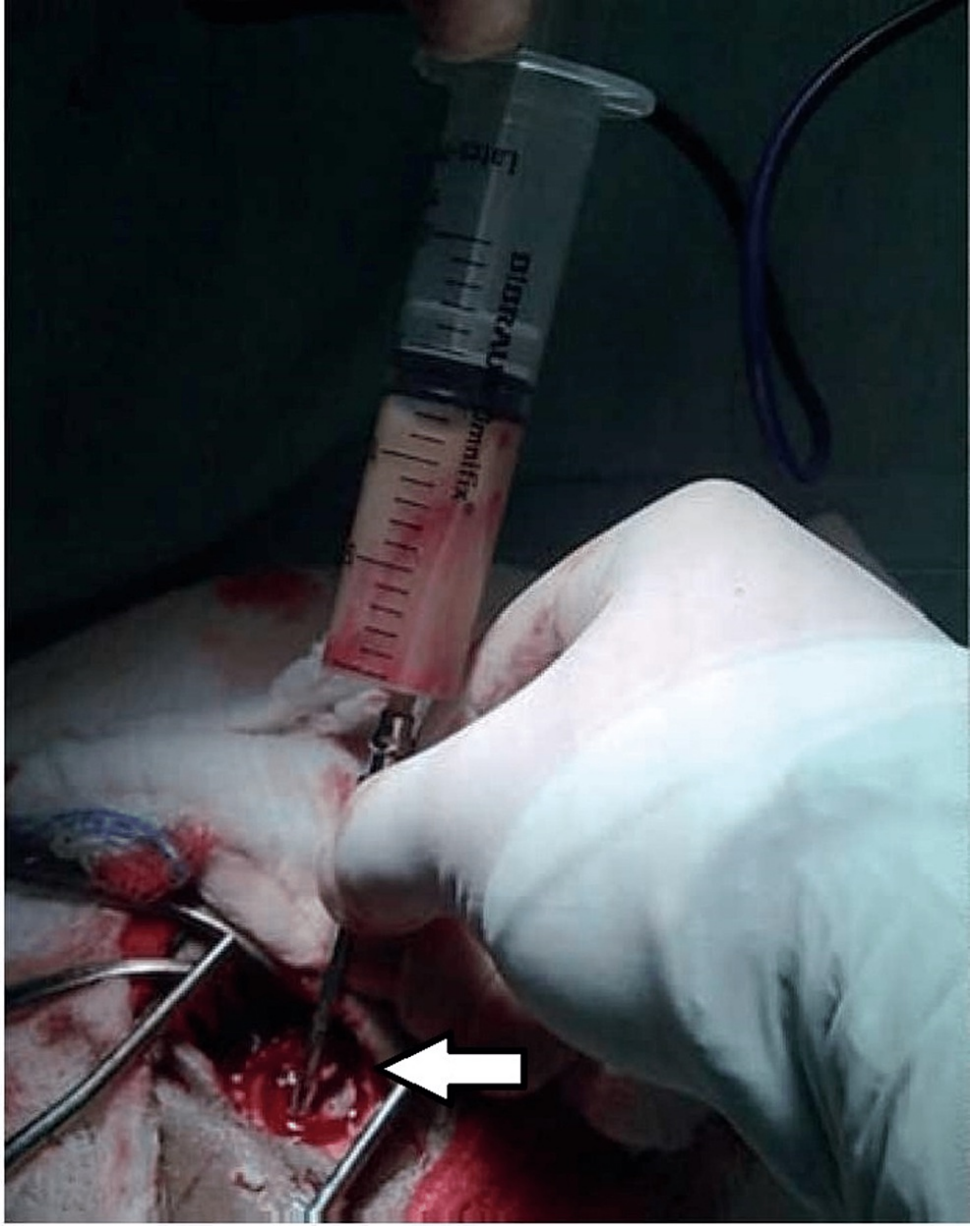
Right otogenic cerebellar abscess drained by burr hole technique

The patient was diagnosed with right chronic otitis media squamosal type with complication (right cerebellar abscess) after taking into account their symptoms, clinical findings, and imaging study results. For the first six days, the patient was prescribed augmentin, metronidazole, and gentamycin. The otolaryngologists and the neurosurgeons worked together as a team to prepare the patient for surgery. Under general anaesthesia, he had a right canal wall down mastoidectomy, and the brain abscess was drained with a bur hole by a neurosurgeon (Figure 4). Intraoperatively, the posterior wall of the external auditory canal was found to be destructed, and cholesteatoma flakes were found in the attic region and the mastoid cavity. The attic wall and mastoid air cells were found to be destroyed. Although the disease appeared to affect the external ear, middle ear, and mastoid and had progressed to result in a cerebellar abscess, it was fascinating to notice that the malleus had only been slightly eroded, and incus and stapes were found to be intact.

The postoperative period for the patient was uneventful. With the culture and sensitivity report supportive of *Pseudomonas aeruginosa*, postoperative antibiotics were altered to ceftazidime, metronidazole, and gentamycin. Anticonvulsant and antioedema measures were taken. A clearing abscess was visible on a contrast-enhanced CT brain scan performed three weeks later. The histopathological analysis supported our clinical and intraoperative findings of cholesteatoma with granulation tissue.

## Discussion

Brain abscess is considered a dreaded complication of otitis media. They do occur and the resulting mortality is still about 10% [2]. It is the infection of the ear and not the reverse as described by Lebert in 1856. It is found to affect the temporal lobe and cerebellum [2]. About 95 % of patients with otogenic brain abscesses were reported with chronic otitis media and cholesteatoma, among which 54% of abscesses were found in the temporal lobe, 44% in the cerebellum, and both locations in 2% of cases. Infection was found to spread outside the confines of the middle ear by extension through demineralization in acute otitis media causing pathological bony defects or bone erosion by cholesteatoma and granulation tissue in COM. Additionally, it was discovered that it might travel through anatomically normal channels, non-anatomical bone flaws brought by injury or neoplastic erosion, and through the periarteriolar spaces of Virchow Robin. In general, the extradural space and venous sinuses are the first areas where infection from the middle ear spreads to neighbouring structures. Meningeal infection results from passage through the dura, and eventually, brain tissue is affected. While cerebellar abscesses are frequently associated with lateral sinus thrombosis, temporal lobe abscesses typically occur after an infection has travelled directly via the tegmen tympani.

Bad-smelling ear discharge, fever, and headache were considered to be the most important early signs and symptoms of neurotologic consequences of chronic otitis media by Schwaber et al. in their review of these symptoms [3]. Muscular incoordination with ataxia, and occasionally coarse spontaneous nystagmus are characteristics of cerebellar abscesses. The clinical characteristics of a brain abscess mirror the stages of the abscess development. In the stage of invasion, the patient presents with headache, low-grade fever, malaise, and drowsiness. While in the stage of localization, the patient is asymptomatic and capsule formation occurs. Abscess enlargement with an edematous zone surrounding it is seen in the stage of enlargement. Raised intracranial pressure, focal symptoms, and signs of disturbance in the brain are also noticed. A growing abscess ruptures into the ventricle or subarachnoid area during the termination stage, causing deadly meningitis. Additionally, the organism involved, the source of the infection (direct extension versus metastasis), the host's immunological condition, the use of corticosteroids, and antibiotic therapy all have an impact on the abscess encapsulation [4].

The most effective diagnostic methods for locating and identifying early abscesses as well as tracking their development have been neuroimaging investigations [3-5]. CT scan is the preferred radiological investigation as it provides useful details on bony erosion of the mastoid and can be used to identify the source of the abscess and the best course of action [6]. Brain abscesses are typically discovered on a brain CT scan in the watershed regions between vascular territories that connect the grey and white matter. These abscesses have smooth, uniform, thin-walled capsules with areas of ring enhancement surrounding hypodense centers [4]. However, MRI provides more information about the abscess than a CT scan does. The imaging technique of choice is contrast-enhanced high-resolution CT, which enables one to identify the size and location of the abscess cavity, distinguish it from other cerebral lesions, and track the clinical course and therapeutic response.

Aspiration, the use of the proper antibiotics, the treatment of after-effects, and the elimination of the primary source are all part of the current approach to treating brain abscesses. Neurosurgical and otolaryngological procedures are often indicated for treating most brain abscesses [2]. The most common surgical treatments for otogenic brain abscess are open mastoidectomy with abscess drained through mastoids (38%), open mastoidectomy alone, and closed mastoidectomy (65%). The patient described here lacked any localizing signs. Suspicion was raised and a CT scan was ordered as a result of the patient's history of headache, dizziness, and vomiting as well as the presence of a fleshy mass, thick greenish discharge in the external auditory canal, and previous history of ear discharge [7]. This made it possible to identify a cerebellar abscess earlier.

## Conclusions

Otogenic brain abscesses are a rare but serious complication of suppurative otitis media. They must be suspected strongly. Otogenic brain abscesses do happen, although the development of antibiotics, better imaging methods, and microsurgical treatment have decreased the frequency of otogenic intracranial problems. For diagnosis and treatment, appropriate imaging studies and multidisciplinary expertise are essential. The cornerstone of efficient management of otogenic brain abscess without complications entails a combined approach between otolaryngologists, neuroradiologists, and neurosurgeons, as well as adequate surgical techniques and apt antimicrobial medication.
